# Knowledge and Practices of the Jeddah Population Towards Tinea Pedis: A Cross-Sectional Study

**DOI:** 10.7759/cureus.86460

**Published:** 2025-06-20

**Authors:** Alhassan H Hobani, Nouf Alhammadi, Ghadi A Shamakhi, Ryof M Sahli, Latifah M Bahkali, Abdulaziz F Zaylaee, Rana Alqahtani, Shahad E Alsulami, Asmaa Aljohani, Hamza A Alamoudi, Rawan M Almoshawer, Razan A Alfakher, Sarah Musaad Albarrak, Sultan Aqeel Jaafari

**Affiliations:** 1 College of Medicine, Jazan University, Jazan, SAU; 2 Rheumatology, King Khalid University, Abha, SAU; 3 College of Medicine, King Khalid University, Abha, SAU; 4 College of Medicine, Jeddah University, Jeddah, SAU; 5 College of Medicine, Taibah University, Al-Madinah al-Munawwarah, SAU; 6 Collage of Medicine, King Abdulaziz University, Jeddah, SAU; 7 School of Medicine, Royal College of Surgeons in Ireland, Dublin, IRL; 8 Collage of Medicine, Imam Abdulrahman Bin Faisal University, Dammam, SAU; 9 College of Medicine, Hail University, Hail, SAU; 10 Dermatology, King Fahad Central Hospital, Jazan, SAU

**Keywords:** awareness, foot examination, hygiene, risk factors, tinea pedis

## Abstract

Introduction: Tinea pedis is a widespread fungal infection that primarily affects the feet, with risk factors including excessive sweating and tight-fitting footwear. Preventive measures, such as maintaining clean and dry feet and wearing sandals in moist environments, are key in preventing its occurrence. Despite its prevalence and preventability, there is limited research on the knowledge and practices related to tinea pedis in Jeddah, Saudi Arabia.

Methodology: A cross-sectional study was conducted through a validated questionnaire among 696 Jeddah residents utilizing Google Forms that were distributed online via social media. Participants were recruited using convenience sampling. Collected data was analyzed using Statistical Software for Social Science (SPSS) version 27.

Results: The results revealed that most participants were unaware of tinea pedis (70.3%) and its risk factors (75.7%) and symptoms (74.6%). Diabetes mellitus was the most frequently selected risk factor, identified by 81.8% of participants from the list provided in the questionnaire. Only 33.3% examined their feet regularly, while 79.5% used nail scissors and 54.7% wore socks regularly. A significant proportion (82.2%) washed their feet three or more times daily. Regular foot examination (odds ratio (OR) = 1.901, p = 0.001) and wearing socks regularly (OR = 1.598, p = 0.027) were significant predictors of awareness, while occupation (unemployed) was also associated with higher odds of awareness (OR = 4.445, p = 0.005). Other factors like age, gender, and education showed no significant association.

Conclusion: Most participants demonstrated significantly poor knowledge of tinea pedis and its risk factors despite its preventable nature. Practices like the regular foot examination and wearing socks were significant predictors of awareness, while lifestyle factors like prolonged sports shoe use and sporadic workouts increased vulnerability. Public health efforts should focus on improving awareness and preventive practices, particularly among high-risk groups such as individuals with diabetes.

## Introduction

Tinea pedis, popularly referred to as athlete's foot, is a common fungal infection that affects feet, and it ordinarily starts between the toes, but can also spread to other parts such as the soles, heels, and the tops of the feet [1]. Globally, tinea pedis is recognized as the most common fungal infection, with an estimated prevalence of 10% in the general population [2]. In Saudi Arabia, tinea pedis is notably prevalent among diabetic patients. In Saudi Arabia, the prevalence is even higher, with a recent study indicating a rate of 15.1% [3]. The burden is not only in high prevalence but also in its potential complications, especially among vulnerable populations such as individuals with diabetes, where it may lead to secondary bacterial infections, foot ulcers, and in severe cases, limb amputation if left untreated [3].

 It is a dermatophyte infection characterized by an itchy, stinging, burning rash on the skin of the feet [2]. In some cases, the infection can also cause the feet to crack and become scaly, often developing a bad odor [2]. The infection is contracted when someone comes into direct contact with the organism, mainly by walking barefoot, but can also be caused by excessively wearing tight-fitting shoes, humid weather, and excessive sweating [3]. Other common risk factors of the infection include obesity, diabetes, and peripheral arterial occlusive disease [3].

Considering the infection being mostly preventable, it is important that people have a sufficient understanding of the risk factors. Some of the simple measures that are highly effective in preventing the infection include maintaining clean and dry feet, limiting time spent in close shoes, wearing sandals, and avoiding walking barefoot, especially in moist environments such as showers [1]. In addition, it is important to seek urgent medical attention whenever one suspects that he or she may be having the infection, as doing so prevents further spread [4]. Seeking medical attention is also essential because if the condition is left untreated, tinea pedis can lead to other comorbidities, such as cellulitis, osteomyelitis, and lymphangitis [5]. Therefore, tinea pedis can significantly affect a person’s health and quality of life, especially if not treated early.

Despite the well-known risk factors and potential impact on the quality of life, there is a lack of studies specifically investigating the knowledge and practices related to tinea pedis among the population of Jeddah, Saudi Arabia. Although Saudi Arabia, particularly its Eastern region near the Arabian Gulf, is considered prone to dermatophyte infections, there remains a significant gap in localized research on public awareness and preventive behaviors in Jeddah [6]. A study by Abanmi et al. found that tinea pedis was the third most common superficial fungal infection (16%) in the Riyadh region, with a prevalence of 11.8% among adults [7]. These findings highlight the importance of assessing the awareness and preventive measures taken by residents of different regions, including Jeddah. However, effective public health initiatives can only be developed with a clear understanding of the current knowledge and practices of the local population. Therefore, this cross-sectional study aims to assess the knowledge and practices of Jeddah residents regarding tinea pedis, providing valuable insights to guide future educational and preventive efforts. The study also examines known risk factors (e.g., diabetes, excessive sweating, tight footwear) and at-risk groups (e.g., individuals with diabetes or those who exercise sporadically).

## Materials and methods

Study design

This online survey targeted adult residents (≥18 years) of Jeddah, Saudi Arabia, recruited via local social media channels. This study adopted a cross-sectional research design that aimed to assess the knowledge and practices of the Jeddah population toward tinea pedis. The study took place between September 2024 and January 2025.

Identification of study participants

This study included individuals aged 18 years and above residing in Jeddah who were willing to participate and independently complete the survey. Participants under 18 years of age or those unable to provide informed consent or complete the survey without assistance were excluded.

Sampling and sample size

The sample size was calculated using Epi Info's formula for population surveys (Centers for Disease Control and Prevention, Georgia, United States). With a 95% confidence level (Z = 1.96), an expected proportion (P) of 50% (to maximize sample size), and a margin of error (E) of 5%, the minimum sample size was 384. However, the sample was increased to 696 to improve on reliability and be more representative. A convenience sampling method was used; data were collected via a self-administered Google Forms (Google LLC, Mountain View, California, United States), distributed online between September 2024 and January 2025. Only respondents confirming residence in Jeddah city (via their registered postal code) were included.

Data collection process

The survey questionnaire was adapted from previous studies conducted in the Taif region that aligned with our study objective [8]. A pilot study with 20 participants was conducted to test reliability and clarity, achieving a Cronbach’s alpha score of 0.781, indicating good internal reliability. These participants involved in the pilot were excluded from the main study. Face validity was conducted by three board-certified dermatologists and two epidemiologists, who assessed each item for clarity, relevance, and cultural appropriateness. The finalized questionnaire was distributed via Google Forms and disseminated across social media platforms, including WhatsApp, Twitter, and Telegram, to maximize reach. The questionnaire consisted of three main sections: one focused on socio-demographic information and the other on knowledge and practices regarding tinea pedis.

Data analysis

Data were entered and cleaned using Microsoft Excel (Microsoft Corporation, Washington, United States), addressing issues such as duplicate entries and missing data. The cleaned dataset was imported into IBM SPSS Statistics for Windows, Version 27.0 (released 2019, IBM Corp., Armonk, NY) for analysis. Descriptive statistics were used to summarize categorical data as frequencies and percentages, and presented as tables. Logistic regression analysis was also performed to identify predictors of good knowledge, like age, gender, education level, and practices of examining feet regularly. A p-value of less than 0.05 is considered statistically significant.

Ethical considerations

The study received ethical approval from King Khalid University Institutional Review Board (IRB) under reference number 2023-1107. Informed consent was obtained from all participants prior to data collection. Participants were assured of confidentiality and anonymity, with the right to withdraw at any stage without penalty. In addition, they were informed of the study’s purpose, potential benefits, and risks.

## Results

Table 1 shows a total of 696 participants involved in the study. Among them, the majority were female (63.2%) and Saudi nationals (91.8%). Nearly half of the respondents (49.6%) were aged between 20 and 30 years, with only 10.8% being above 60 years. Regarding education, the largest group consisted of university graduates (63.1%), followed by those with secondary education (23.1%). In terms of occupation, students made up the majority (50.4%), while 22.4% were categorized under other professions. Most participants had a BMI within the normal range (18.5-24.9, 43.1%), although 24.1% were classified as obese (BMI > 30). In addition, the vast majority (85.6%) of participants were non-smokers.

**Table 1:** Sociodemographic information of the participants (n = 696). Sociodemographic variables presented as counts (n) and frequencies (%).

Sociodemographic variables	Category	Frequency (n) and proportions (%)
Gender	Female	440 (63.2%)
	Male	256 (36.8%)
Age	Below 20 years	69 (9.9%)
	20-30 years	345 (49.6%)
	31-40 years	80 (11.5%)
	41-50 years	68 (9.8%)
	51-60 years	59 (8.5%)
	Above 60 years	75 (10.8%)
Nationality	Saudi	639 (91.8%)
	Non-Saudi	57 (8.2%)
Education	Primary	41 (5.9%)
	Secondary	161 (23.1%)
	University	439 (63.1%)
	Post-graduate	55 (7.9%)
Occupation	Student	351 (50.4%)
	Teacher	89 (12.8%)
	Military	1 (0.1%)
	Housewife	23 (3.3%)
	Retired	6 (0.9%)
	Engineer	47 (6.8%)
	Doctor	23 (3.3%)
	Others	156 (22.4%)
BMI	Below 18.5	74 (10.6%)
	18.5- 24.9	300 (43.1%)
	25- 29.9	154 (22.1%)
	More than 30	168 (24.1%)
Smoking status	Smoker	100 (14.4%)
	Non-smoker	596 (85.6%)

Table 2 summarizes various foot hygiene practices among the participants. The majority, 464 (66.7%), reported not examining their feet regularly. However, an overwhelming 553 (79.5%) stated that they regularly use nail scissors. Slightly more than half, 381 (54.7%), indicated that they wear socks regularly, with most participants, 470 (67.5%), wearing socks predominantly during the winter season. Regarding foot washing frequency, a substantial 572 (82.2%) reported washing their feet three times or more daily, while only 124 (17.8%) washed their feet less than three times a day. 

**Table 2:** Foot hygiene practices among the participants Data are presented as counts (n) and frequencies (%).

Variables	Category	Count (%)
Do you routinely check your feet?	Yes	232 (33.3%)
	No	464 (66.7%)
Do you consistently use nail scissors?	Yes	553 (79.5%)
	No	143 (20.5%)
Do you wear socks regularly?	Yes	381 (54.7%)
	No	315 (45.3%)
Wearing socks	Rarely	226 (32.5%)
	During winter	470 (67.5%)
How often do you wash your feet each day?	At least three times a day	572 (82.2%)
	Less than three times	124 (17.8%)

Table 3 depicts that the majority, 487 (70.3%), were not aware of tinea pedis, and 527 (75.7%) were unaware of its risk factors. Among the identified risk factors, diabetes mellitus was the most prevalent, reported by 569 (81.8%) participants. Similarly, most participants, 519 (74.6%), were not aware of the symptoms of tinea pedis, although pain was the most commonly recognized symptom, noted by 477 (68.5%) respondents. Regarding physical activity, 239 (34.3%) reported working out sometimes, and 219 (31.5%) indicated wearing sports shoes for four to eight hours daily, which was the most frequent duration of shoe usage. 

**Table 3:** Respondents' knowledge about tinea pedis Awareness information are presented as counts (n) and frequencies (%).

Variables	Category	Frequency (n) and proportions (%)
Are you aware of tenia pedis?	Yes	207 (29.7%)
	No	487 (70.3%)
Are you aware of tenia pedis risk factors?	Yes	169 (24.3%)
	No	527 (75.7%)
Risk factors of tenia pedis include	Diabetes mellitus (DM)	569 (81.8%)
	HIV	197 (28.3%)
	Peripheral arterial disease	221(31.8%)
Are you aware of tenia pedis symptoms?	Yes	177 (25.4%)
	No	519 (74.6%)
Symptoms of tenia pedis include	Pain	477 (68.5%)
	Itching	333 (47.8%)
	Redness	346 (49.7%)
Do you do workouts?	Never	98 (14.1%)
	Little	197 (28.1%)
	Sometimes	239 (34.3%)
	Usually	116 (16.7%)
	Always	46 (6.6%)
How often do you wear sports shoes?	Less than 2 hours	177 (25.4%)
	2-4 hours	207 (29.7%)
	4-8 hours	219 (31.5%)
	More than 8 hours	93 (13.4%)

Table 4 shows that while most occupations did not significantly impact awareness, unemployed individuals had significantly higher odds of awareness compared to the other groups (OR = 4.445, 95% CI: 1.585-12.470, p = 0.005). Behavioral factors, however, emerged as key predictors. Participants who regularly examined their feet were nearly twice as likely to be aware of tinea pedis (OR = 1.901, 95% CI: 1.310-2.759, p = 0.001). Similarly, wearing socks regularly was associated with significantly higher odds of awareness (OR = 1.598, 95% CI: 1.054-2.422, p = 0.027). The use of nail scissors did not significantly impact awareness (OR = 1.438, 95% CI: 0.843-2.453, p = 0.182). Gender did not show a significant effect, with males having slightly lower odds of awareness compared to females (OR = 0.882, 95% CI: 0.585-1.330, p = 0.550). Other sociodemographic variables like age, nationality, and education also did not influence awareness. 

**Table 4:** Logistic regression analysis of factors influencing awareness of tinea pedis among the participants Logistic regression of foot-hygiene practices as predictors of good tinea pedis knowledge (crude OR, 95% CI).

Variable	Category	Coef (B)	SE	Crude odds ratio (Exp B)	95% CI	P-value
Gender	Male	-0.125	0.209	0.882	(0.585, 1.330)	0.55
	Female (Ref)			1		
Nationality	Saudi	0.013	0.349	1.013	(0.511, 2.009)	0.969
	Non-Saudi (Ref)			1		
Education	Illiterate	-0.304	0.639	0.738	(0.211, 2.580)	0.634
	Primary	0.318	0.407	1.375	(0.619, 3.053)	0.434
	Secondary	0.181	0.371	1.198	(0.578, 2.481)	0.627
	University/postgraduate (Ref)			1		
Age	<20	-0.558	0.524	0.573	(0.205, 1.599)	0.287
	20–30	0.052	0.423	1.053	(0.459, 2.415)	0.902
	31–40	0.045	0.429	1.046	(0.451, 2.423)	0.917
	41–50	-0.446	0.459	0.64	(0.261, 1.574)	0.331
	51–60	0.081	0.43	1.085	(0.467, 2.521)	0.85
	>60 (Ref)			1		
Occupation	Retired	0.349	0.33	1.418	(0.742, 2.709)	0.29
	Military	-18.104	0	1.37E-08	(1.373E-08, 1.373E-08)	0.999
	Unemployed	1.492	0.526	4.445	(1.585, 12.470)	0.005*
	Other (Ref)			1		
Do you examine your feet regularly?	Yes	0.642	0.19	1.901	(1.310, 2.759)	0.001*
	No (Ref)			1		
Do you use nail scissors regularly?	Yes	0.363	0.272	1.438	(0.843, 2.453)	0.182
	No (Ref)			1		
Do you wear socks regularly?	Yes	0.469	0.212	1.598	(1.054, 2.422)	0.027*
	No (Ref)			1		
BMI	<18.5	0.573	0.327	1.774	(0.935, 3.367)	0.08
	18.5–24.9	0.189	0.239	1.208	(0.757, 1.929)	0.428
	25–29.9	0.084	0.275	1.087	(0.635, 1.863)	0.76
	≥30 (Ref)			1		
How many times do you wash your feet daily?	1	0.32	0.26	1.377	(0.826, 2.293)	0.22
	≥2 (Ref)			1		

## Discussion

Tinea pedis is a common fungal infection with significant complications globally, affecting both individuals with compromised immunity and the general population [9]. This study aimed to assess the level of knowledge and practices regarding tinea pedis among the Jeddah population. Despite its prevalence as a well-recognized fungal infection, a majority of the participants, 487 (70.3%), were unaware of tinea pedis. Furthermore, an even larger proportion, 527 (75.7%), were unaware of the risk factors associated with the condition, reflecting a low level of knowledge within this population. These findings align with global studies reporting similarly low awareness levels. For example, a study conducted by Jaishi et al. in Nepal found that only 20% of participants were familiar with risk factors for fungal infections [10]. Similarly, research by Khalif et al. in Saudi Arabia revealed that 70% of individuals with tinea pedis were unaware of its symptoms until they experienced disease manifestation [11].

The study further tried to unearth more about the participants’ practices on measures promoting foot hygiene. A majority of respondents, 464 (66.7%), reported that they did not regularly examine their feet, highlighting a potential gap in adherence to preventive measures among the participants. However, a significant proportion, 553 (79.5%), reported using nail scissors regularly, which reflects good hygiene practices. These findings contrast with a study conducted in Saudi Arabia by Alqahtani et al., which found that 71.5% of diabetic patients regularly examined their feet and toes as a precautionary measure to monitor for tinea pedis [12]. This supports the expression of a majority of respondents 527 (75.7%) who have identified DM as the major risk factor of tinea pedis in this present study; moreover, a study by Marghani et al carried out in Bahrain established that only 45% of diabetic foot ulcers patients examined their feet regularly portraying inconstancy of foot public health care practices [13]. These disparities in practices could be attributed to differences in public health campaigns, healthcare access, or cultural attitudes toward personal health monitoring.

Several lifestyle practices are associated with tinea pedis. This study unveiled that a substantial proportion of respondents, 219 (31.5%), wore sports shoes for between four and eight hours. Although not scientifically proven, previous studies have established that this habit creates a favorable environment for the magnification of fungi. A study by Sasagawa et al. has attested that prolonged wear of sports shoes generates comfy conditions beneficial for fungal procreation [14]. In addition, more than a third of respondents, 239 (34.3%), reported engaging in workouts only occasionally. This sporadic activity, coupled with sweating during exercise, could increase their susceptibility to tinea pedis, as sweating creates a moist environment that promotes fungal growth. This observation is supported by Leung et al., who found that excessive sweating during physical activities is a significant risk factor for tinea pedis [1].

In this study, behavioral practices emerged as crucial indicators of tinea pedis knowledge among participants. Regular examination of feet (OR = 1.901, p = 0.001) was a strong predictor of awareness, indicating that individuals who examined their feet regularly were 1.9 times more likely to be aware of tinea pedis compared to those who did not. Similarly, wearing socks regularly (OR = 1.598, p = 0.027) was another significant factor, showing that participants who wore socks regularly were 1.6 times more likely to be aware of tinea pedis. These findings align with a study by Denning et al., which established a statistically significant association between certain foot hygiene practices and awareness of tinea pedis [15]. Interestingly, the use of nail scissors, while a common hygiene practice, did not significantly predict awareness of tinea pedis (p = 0.182), suggesting that not all hygiene behaviors directly correlate with knowledge of tinea pedis. In terms of sociodemographic variables, occupation was the only factor significantly associated with tinea pedis awareness, while other variables, such as age and gender, showed no statistically significant relationship.

One of the key limitations of this study was that the study's cross-sectional design limits its ability to establish a causal relationship between behaviors, lifestyle factors, and tinea pedis awareness or prevalence. In addition, because data were collected via an online Google Forms, selection and response bias may have occurred (e.g., over-representation of younger, more tech-savvy individuals), which could limit the generalizability of findings to all Jeddah residents. Longitudinal studies are necessary to confirm cause-and-effect relationships and to explore potential temporal associations between specific risk factors, such as prolonged footwear usage and physical activity patterns, with the development of tinea pedis.

## Conclusions

In conclusion, the majority of participants demonstrated limited knowledge of tinea pedis and its associated risk factors. Behavioral practices, such as regular foot examination and wearing socks, emerged as strong predictors of knowledge and lifestyle factors, such as prolonged wearing of sports shoes and sporadic workout routines. Hence, there is a need for public health initiatives to address both awareness and preventive practices, particularly for at-risk groups such as individuals with diabetes, who were identified by participants as being highly vulnerable to tinea pedis.

## Appendices

**Table 5:** Sociodemographic information of the participants

Social Demographic Variables	Category
Gender	Female
	Male
Age	Below 20 years
	20 -30 years
	31-40 years
	41- 50 years
	51- 60 years
	Above 60 years
Nationality	Saudi
	Non- Saudi
Education	Primary
	Secondary
	University
	Post-graduate
Occupation	Student
	Teacher
	Military
	Housewife
	Retired
	Engineer
	Doctor
	Others
BMI	Below 18.5
	18.5- 24.9
	25- 29.9
	More than 30
Smoking Status	Smoker
	Non- Smoker

**Table 6:** Foot Hygiene Practices Among the Participants

Variables	Category
Do you routinely check your feet?	Yes
	No
Do you consistently use nail scissors?	Yes
	No
Do you wear socks regularly?	Yes
	No
Wearing socks	Rarely
	During Winter
How often do you wash your feet each day?	At least three times a day
	Less than three times

**Table 7:** Respondents Awareness About Tinea pedis

Variables	Category
Are you aware of Tenia pedis?	Yes
	No
Are you aware of Tenia pedis risk factors?	Yes
	No
Risk factors of Tenia pedis include	Diabetes Mellitus( DM)
	HIV
	Peripheral Arterial Disease
Are you aware of Tenia pedis symptoms?	Yes
	NO
Symptoms of Tenia Pedis include	Pain
	Itching
	Redness
Do you do workouts?	Never
	Little
	Sometimes
	Usually
	Always
How often do you wear sport shoes?	Less than 2 hours
	2- 4 hours
	4- 8 hours
	More than 8 hours
